# Supplementary figures and images for: ColocZStats: a z-stack signal colocalization extension tool for 3D slicer
Source: Front Physiol. 2024 Sep 4;15:1440099. doi: 10.3389/fphys.2024.1440099 (PMC11408364; doi:10.3389/fphys.2024.1440099)

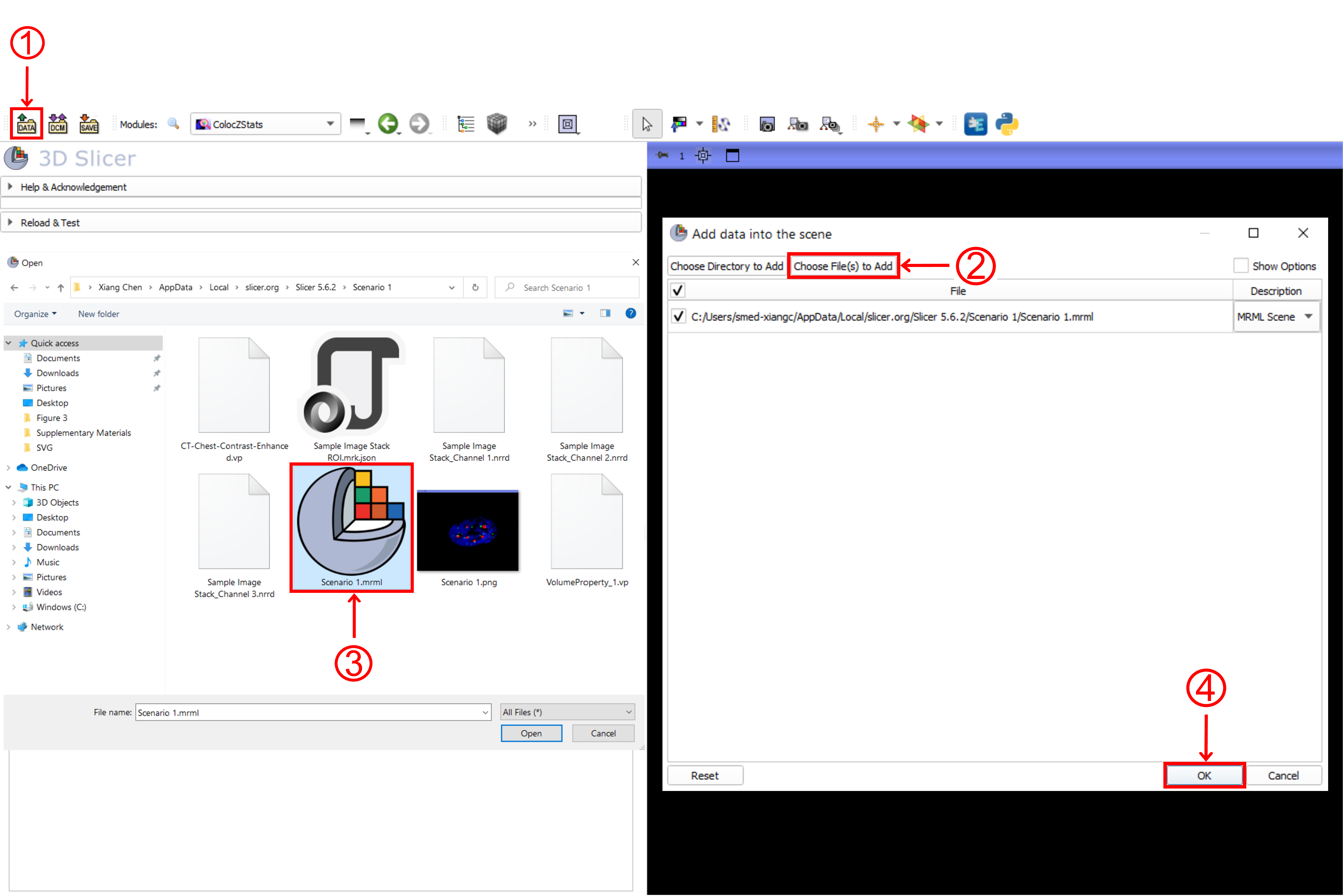

Supplement: Supplementary file 1 [file Image3.TIFF]

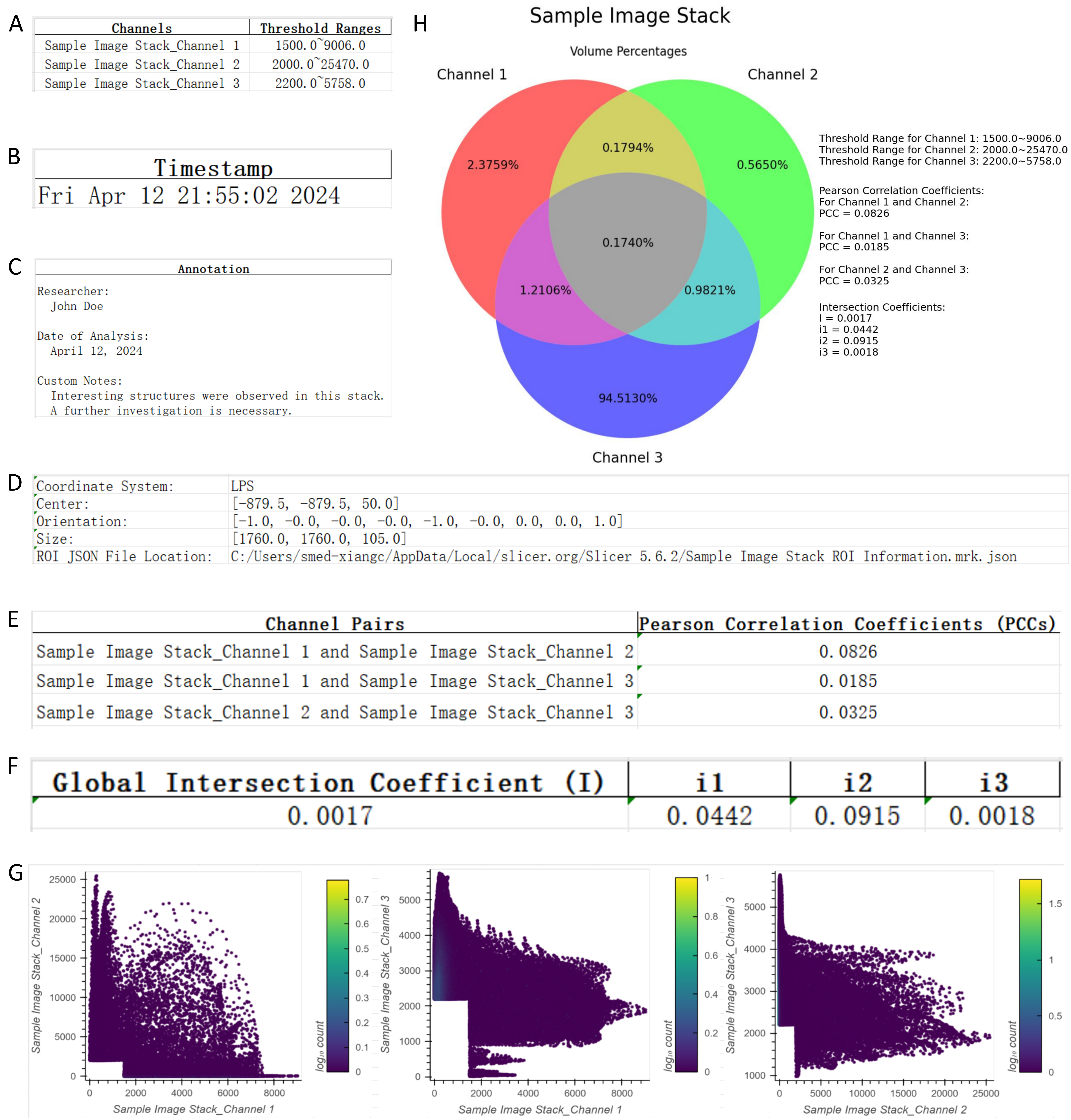

Supplement: Supplementary file 3 [file Image1.TIFF]

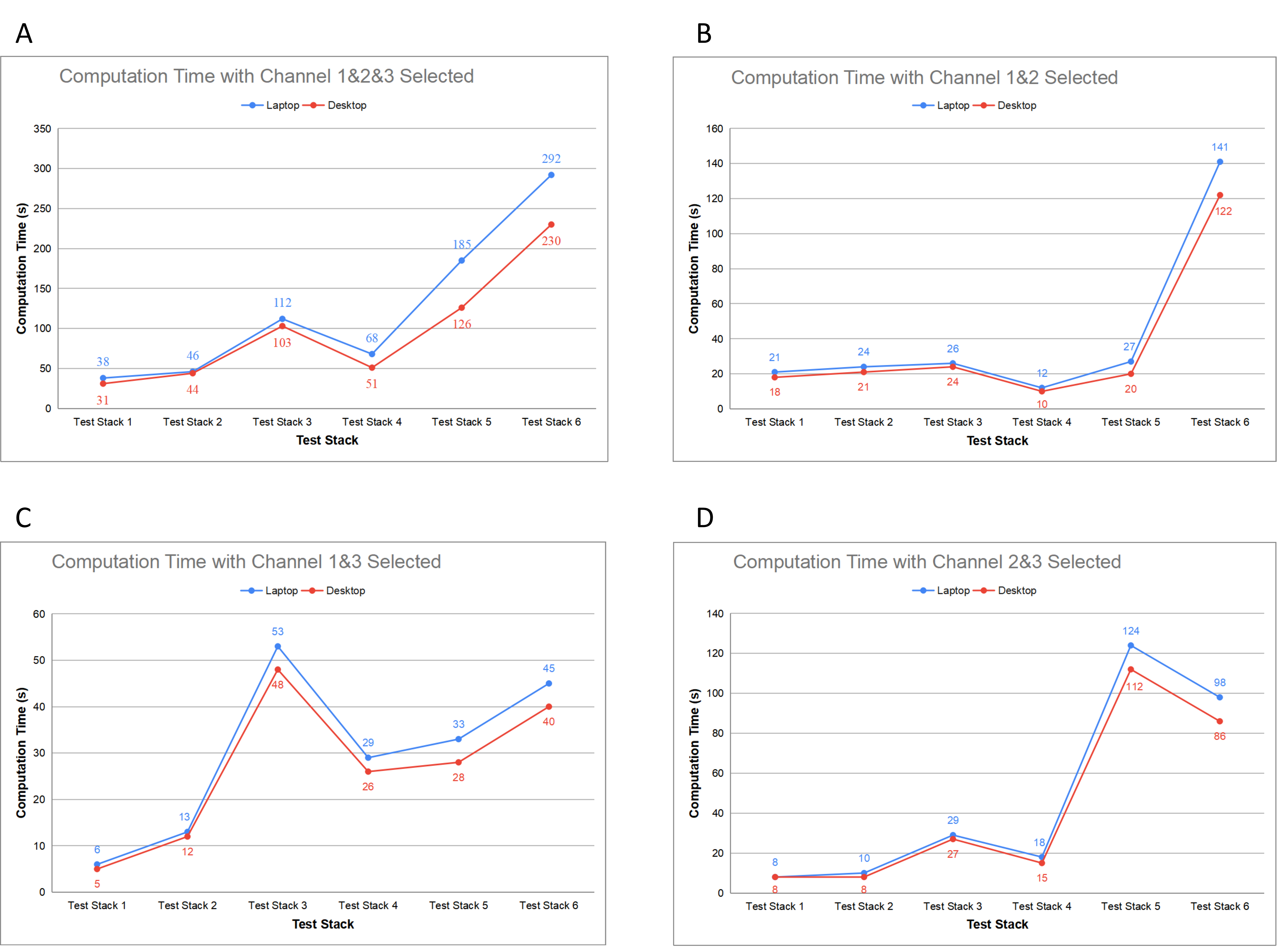

Supplement: Supplementary file 4 [file Image5.TIFF]

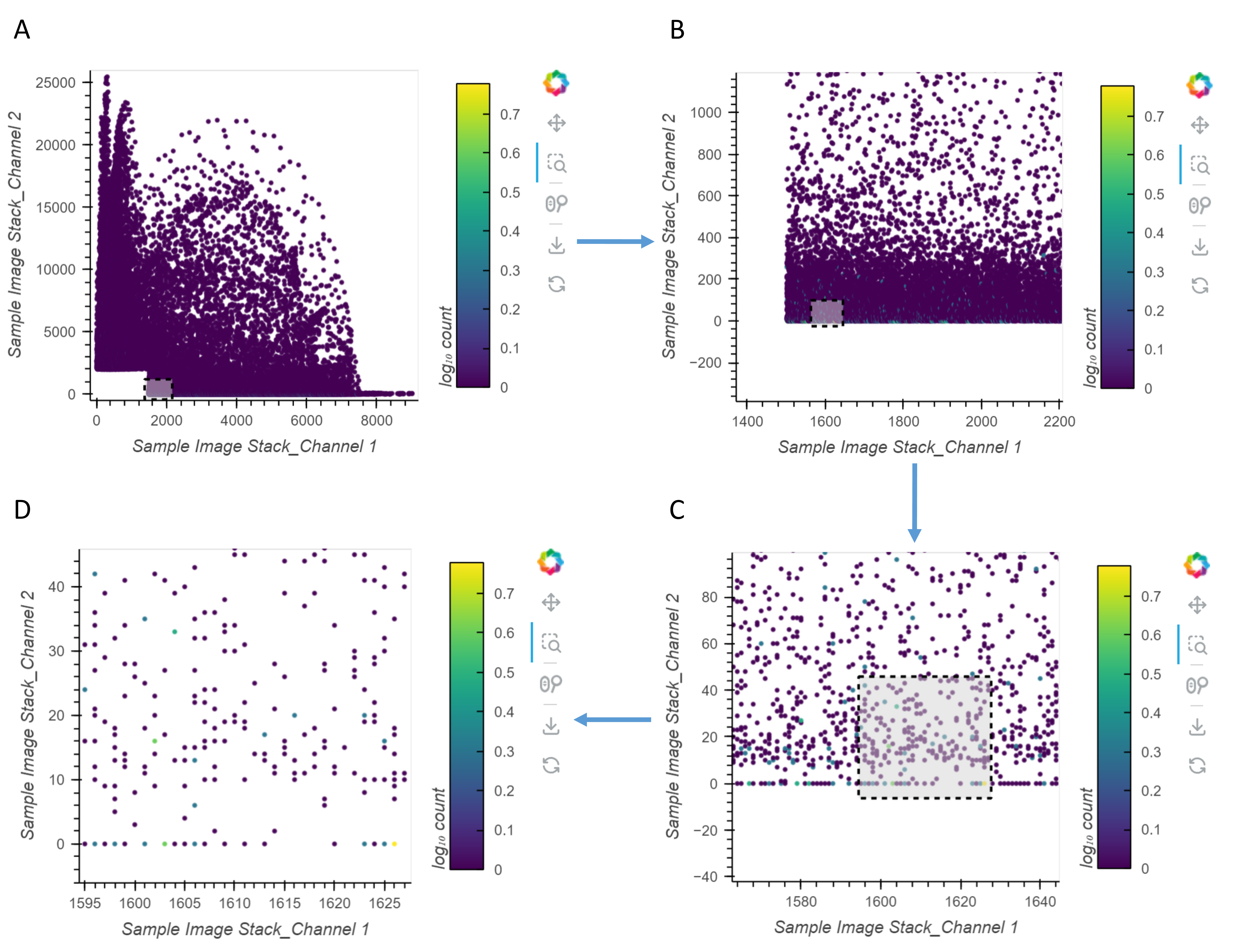

Supplement: Supplementary file 5 [file Image2.TIFF]

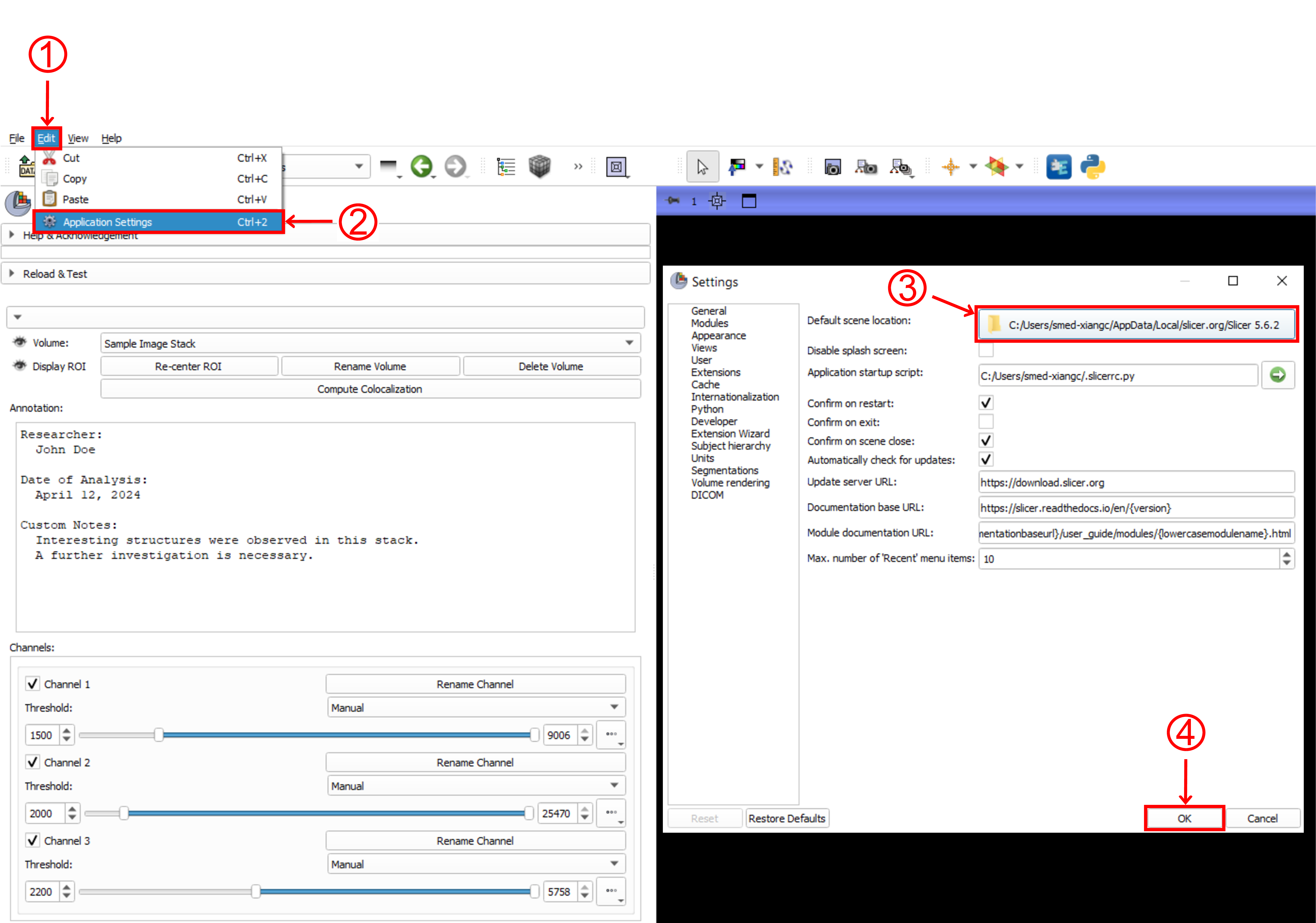

Supplement: Supplementary file 6 [file Image4.TIFF]
