# Supplementary material for: ColocZStats: a z-stack signal colocalization extension tool for 3D slicer
Source: Front Physiol. 2024 Sep 4;15:1440099. doi: 10.3389/fphys.2024.1440099 (PMC11408364; doi:10.3389/fphys.2024.1440099)
Supplement: Supplementary file 2 [file DataSheet2.pdf]

Supplementary Material

1 TECHNICAL SPECIFICATIONS OF THE SAMPLE IMAGE STACKS

As mentioned in section 2.1 of the article, the ‘Sample Image Stack.tif’ is the confocal z-stack data that we predominantly utilized, and it was collected during a study on the colocalization of DSS1 nuclear bodies with other nuclear body types. More specifically, a human ovarian clear cell carcinoma cell line (RMG-I) was seeded at 100,000 cells per well onto a coverslip in a 6-well plate and allowed to grow till 70% confluency. On the day of staining, the cells were fixed with 4% ice-cold paraformaldehyde for 15 mins at room temperature (RT) and blocked with 2% Bovine Serum Albumin (BSA) in 0.1% Phosphate Buffer Saline containing 0.1% Triton-X (PBSTx) for 30 mins. Following fixation and blocking, the cells were incubated with a primary antibody cocktail containing anti-DSS1 (Catalogue# NB100-1334, Novus Biologicals) and anti-PML (Catalogue#sc-966, SCBT) for 1h at RT. After that, the cells were washed 3 times with 0.1% PBSTx for 5 mins each and incubated with a secondary antibody cocktail containing anti-goat Alexa Fluor^{TM} 647 (for DSS1), anti-mouse Alexa Fluor^{TM} 488 (for PML) and Hoechst 33342 (Catalogue# H3570, Invitrogen) for 1h at RT. Following this incubation, the cells were subjected to 3 washes, each lasting 5 mins, with 0.1% PBSTx to ensure thorough cleansing. Moreover, this stack show colocalization of DSS1 nuclear bodies (Red, 647 channel) with PML nuclear bodies (Green, 488 channel) in RMG-I cell line. Cellular nucleus (Blue, 405 channel) was stained using Hoechst 33342 (see Table S1). Also, the metadata information for this stack and the additional stack used in section 4.8 of the article are summarized in Table S2.

In Table S2, ‘Sample Stack 1’ refers to the ‘Sample Image Stack.tif,’ and ‘Sample Stack 2’ refers to the additional stack, ‘Airyscan_Calnexin A20_Z_01-Airyscan Processing.tif.’ The objective lens used for both acquisitions was a Zeiss Plan-Apochromat Oil Objective 63x. The software utilized for both acquisitions is ZEISS LSM800 Zen (Black Edition), which has the capability to advise users to be able to select the optimal scan format and frame size based on the Nyquist criterion before image capture, thus preventing undersampling or oversampling during the image acquisition process. This is why the pixel size used for image acquisition matches the one calculated by the Nyquist criterion. Additionally, both acquisitions were sequentially scanned, with one channel/detector scanned after another one. This is the baseline precaution that will avoid cross-talk and bleed-through.

Furthermore, it is possible to envision the use of Bio-Formats in ImageJ in the future, to help eliminate the painful step of exporting TIFF images and generate more data to be stored. Bio-Formats is a software that applies standardized, open formats for reading and writing image data. More specifically, it is a community-driven project that provides a standardized application interface, capable of reading more than 140 proprietary file formats such as Carl Zeiss Czi, Leica Lif, Olympus Oif, and Nikon Nd and convert them to an open, OME-based model that any software can read.

Target	Primary Antibody	Secondary Antibody	Confocal Channel
**DSS1**	Anti-DSS1 Cat# NB100-1334, Novus Biologicals	Anti-goat Alexa Fluor^{TM} 647 Cat# A-21447, Invitrogen	647
**PML**	Anti-PML Cat#sc-966, SCBT	Anti-mouse Alexa Fluor^{TM} 488 Cat# A-11059, Invitrogen	488
**Nucleus**	Hoechst 33342 Cat# H3570, Invitrogen		405

Table S1. Immunocytochemistry

Metadata / Stacks	Objective Numerical Aperture (NA)	Pixel Size (Image Acquisition)	Pixel Size (Nyquist Criterion)	Image Acquisition Software	Sampling Step in Z	Airyscan Function	Sampling in X, Y, Z (Super-resolution)
**Sample Stack 1**	1.4	28 nm x 28 nm	28 nm x 28 nm	ZEISS LSM800 Zen (Black Edition)	0.15 µm	√	28 nm x 28 nm x 150 nm (L,w,h of per pixel)
**Sample Stack 2**	1.4	28 nm x 28 nm	28 nm x 28 nm	ZEISS LSM800 Zen (Black Edition)	0.15 µm	√	28 nm x 28 nm x 150 nm (L,w,h of per pixel)

Table S2. Metadata Information of Stacks in the Article

2 DESCRIPTION OF MRML FILES

All loaded data in 3D Slicer can be stored in a data repository called the ‘MRML scene’ (MRML stands for ’Medical Reality Modeling Language’). All those graphical representations and statistical coefficients generated by ColocZStats, as shown in sections 4.6 and 4.8 of the article, can be obtained by loading corresponding files with the ‘.mrml’ file extension into the current stable version of 3D Slicer (V5.6.2). In the supplementary materials, there are three folders containing MRML files, each of which is archived within a separate zip file.

Specifically, the MRML file stored in ‘Scenario 1.zip’ can be utilized to generate all result illustrations and statistical coefficients corresponding to section 4.6 in the article. The MRML files saved in ‘Scenario 2.zip’ can be used to produce the relevant illustrations presented in the first two rows of Figure 8 in Section 4.8. The MRML files saved in ‘Scenario 3.zip’ can be used to produce the relevant illustrations presented in the last two rows of Figure 8 in Section 4.8.

Additionally, an example results spreadsheet (Sample Image Stack Statistics.xlsx) is included in the supplementary materials, and Figure S1 showcases all of its sub-sheets. Among them, Figure S1D displays some crucial information related to the defined ROI. The information is automatically and selectively extracted from a JSON file containing all details of the ROI node, and the JSON file is for reloading the ROI box. By invoking the 3D Slicer's built-in exporting method for such JSON files in ColocZStats' background process, the JSON file will also be automatically saved after each calculation. Notably, the JSON file can also be saved as one of the batch files produced by clicking the 'SAVE' button of 3D Slicer. All the files included in scenario zips are the batch files generated this way, and the MRML files refer to all the other batch files in their respective folder for reloading operation scenarios.

Moreover, the generated 2D histograms will be automatically saved as static images and as corresponding HTML files, which include several interactive options for viewers to examine the data in greater detail. As shown in Figure S2, viewers can select the 'magnifier' option on the right to zoom into the 2D histogram iteratively, getting to an ever closer look at the data, and this may reveal items with different values (and correspondingly different colors) after zooming in.

The example HTML file has been saved in an individual zip file (Sample 2D Histogram HTML.zip) and is provided in the supplementary materials, whereas the sample image z-stack and scenario files can be found at the Zenodo repository 10.5281/zenodo.13219935

3 STEPS TO LOAD MRML FILES

Before loading any MRML files into 3D Slicer 5.6.2, a necessary step is installing ColocZStats. For detailed instructions on how to install ColocZStats, please see its repository homepage on GitHub.

Taking 'Scenario 1.zip' as an example, please follow the steps below to load the MRML files:

1. Unzip 'Scenario 1.zip' to get a folder with the same name.
2. Please follow the numerical order as indicated in Figure S3 to load the 'Scenario 1.mrml' file from the folder obtained in the first step into the scene.

Upon loading any unzipped MRML files into 3D Slicer, all of its corresponding GUI elements and volume renderings will appear. Following this, please use the control panel of ColocZStats to select the channels and threshold ranges to be analyzed. Then, click the 'Compute Colocalization' button to generate the corresponding Venn diagrams, 2D histograms, and coefficient results described in the article's respective sections. Simultaneously, all the generated diagrams and results spreadsheet will be automatically saved to the 'Default scene location' of 3D Slicer. Figure S4 illustrates how to specify the 'Default scene location' in 3D Slicer. By default, the 'Default scene location' is the installation location of 3D Slicer.

4 COMPUTER REQUIREMENTS AND PERFORMANCE ANALYSIS

ColocZStats can be used with a variety of computer systems. To illustrate the performance under different computer systems, we selected a portable platform (laptop) and a desktop platform. Then, we selected several image stacks of different sizes to evaluate the computation time under both platforms.

The configuration details of the two computer systems we used are summarized in table S3.

We first selected six image stacks of different sizes for the test of computation time. Subsequently, we installed the same version of 3D Slicer and ColocZStats on the two computer systems. Each stack was

Specs / Devices	System	GPU	CPU	Installed RAM
**Laptop**	Windows 10 Enterprise 22H2	NVIDIA GeForce RTX 2060	Intel(R) Core(TM) i7-10750H, 2.60GHz, 2592Mhz, 6 Core(s), 12 Logical Processor(s)	16.0 GB (15.8 GB usable)
**Desktop**	Windows 10 Pro 22H2	NVIDIA GeForce RTX 4070 Ti	AMD Ryzen 9 7900X, 4.70GHz, 4701Mhz, 12 Core(s), 24 Logical Processor(s)	64.0 GB (63.1 GB usable)

Table S3. Configuration Details Summary

then sequentially imported into ColocZStats on each device. Following this, we recorded the computation time for each stack in ColocZStats on each device for all possible channel selection cases. Notably, these stacks each contain at least three channels. For each stack, four channel selection cases are included in this description for demonstration purposes: Channel 1&2&3 selected; Channel 1&2 selected; Channel 1&3 selected; and Channel 2&3 selected. As a result, four corresponding line charts were generated. Additionally, for each channel selection case, all stacks were analyzed entirely under the full threshold ranges of their selected channels. Figure S5 illustrates the resulting line charts showing the computation times for analyzing all test stacks on each device with each channel selection case. The above tests show that the computation time may vary depending on the size of the loaded image stack, the number of voxels within the ROI box, or the specific computer configuration. For stacks within 1GB in size, when the entire stack is selected and the threshold ranges for each channel are set to the full range, obtaining all result illustration diagrams and spreadsheets generally takes a few seconds to a few minutes. All the test z-stacks used here can be found at the same Zenodo repository 10.5281/zenodo.13219935.

A

Channels	Threshold Ranges
Sample Image Stack_Channel 1	1500.0~9006.0
Sample Image Stack_Channel 2	2000.0~25470.0
Sample Image Stack_Channel 3	2200.0~5758.0

B

Timestamp
Fri Apr 12 21:55:02 2024

C

Annotation
Researcher:<br>John Doe<br><br>Date of Analysis:<br>April 12, 2024<br><br>Custom Notes:<br>Interesting structures were observed in this stack.<br>A further investigation is necessary.

H

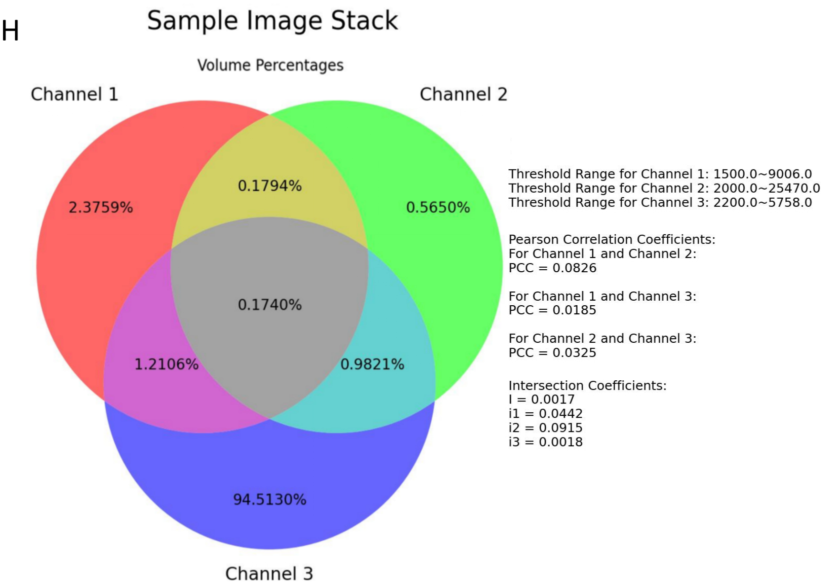

D

Coordinate System:	LPS
Center:	[-879.5, -879.5, 50.0]
Orientation:	[-1.0, -0.0, -0.0, -0.0, -1.0, -0.0, 0.0, 0.0, 1.0]
Size:	[1760.0, 1760.0, 105.0]
ROI JSON File Location:	C:/Users/smed-xiangc/AppData/Local/slicer.org/Slicer 5.6.2/Sample Image Stack ROI Information.mrk.json

E

Channel Pairs	Pearson Correlation Coefficients (PCCs)
Sample Image Stack_Channel 1 and Sample Image Stack_Channel 2	0.0826
Sample Image Stack_Channel 1 and Sample Image Stack_Channel 3	0.0185
Sample Image Stack_Channel 2 and Sample Image Stack_Channel 3	0.0325

F

Global Intersection Coefficient (I)	i1	i2	i3
0.0017	0.0442	0.0915	0.0018

G

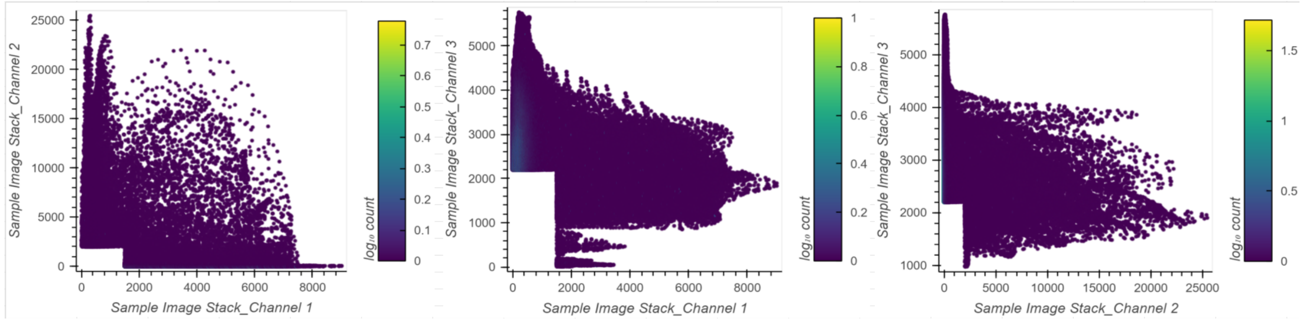

Figure S1. All of the sub-sheets in each spreadsheet result from each single computation. **(A)** The selected channels and their respective threshold ranges. **(B)** The timestamp of this computation. **(C)** The custom annotation for the specified image stack. **(D)** The related information of the defined ROI for this computation. **(E)** The PCCs for all possible pairwise combinations of the selected channels. **(F)** All the resulting intersection coefficients. **(G)** All the resulting 2D histograms, with each histogram illustrating a pair of channels. **(H)** The resulting illustration embedded with the Venn diagram and all coefficient results.

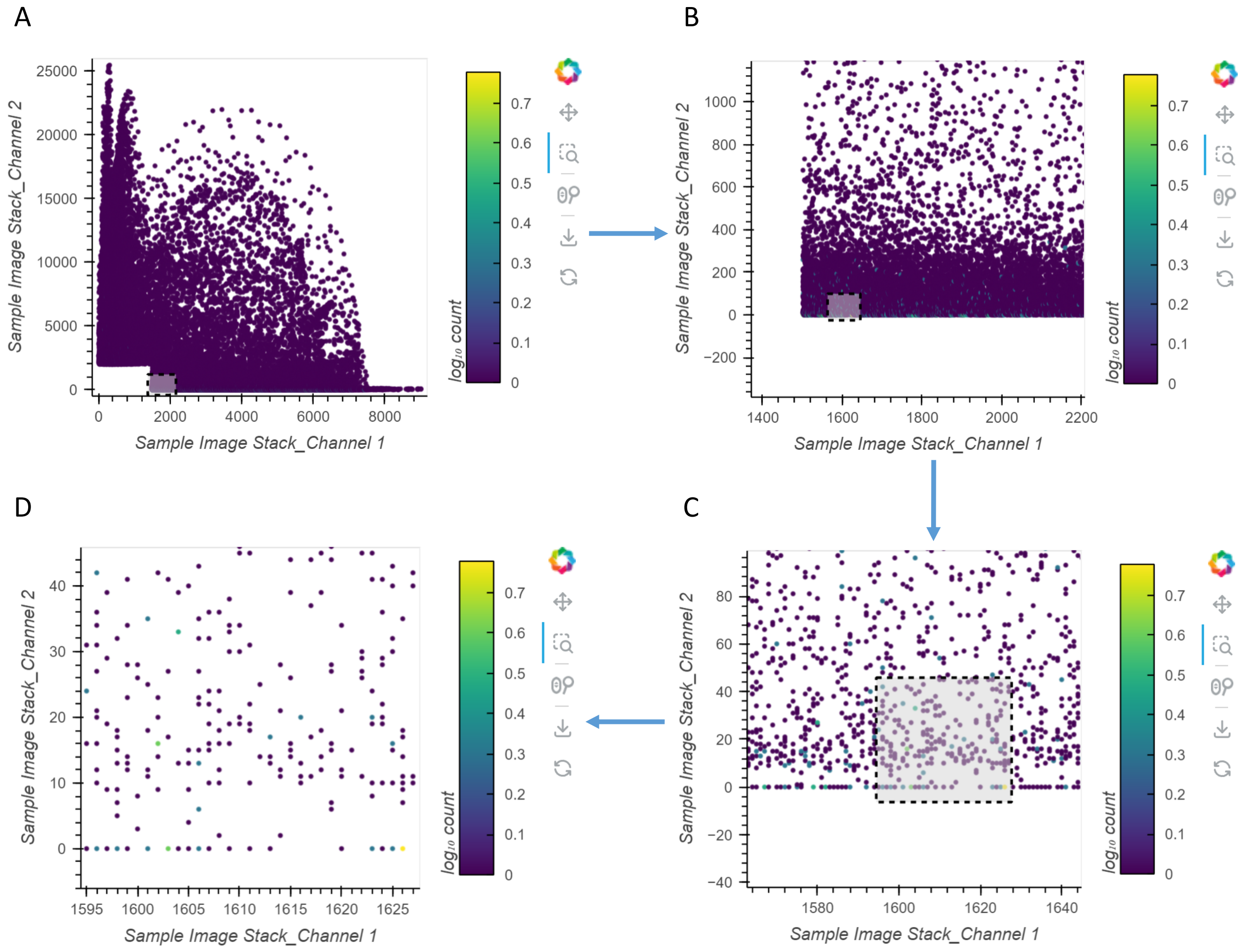

Figure S2. The sample 2D histogram's interactive HTML. Viewers can select any area to zoom into it iteratively.

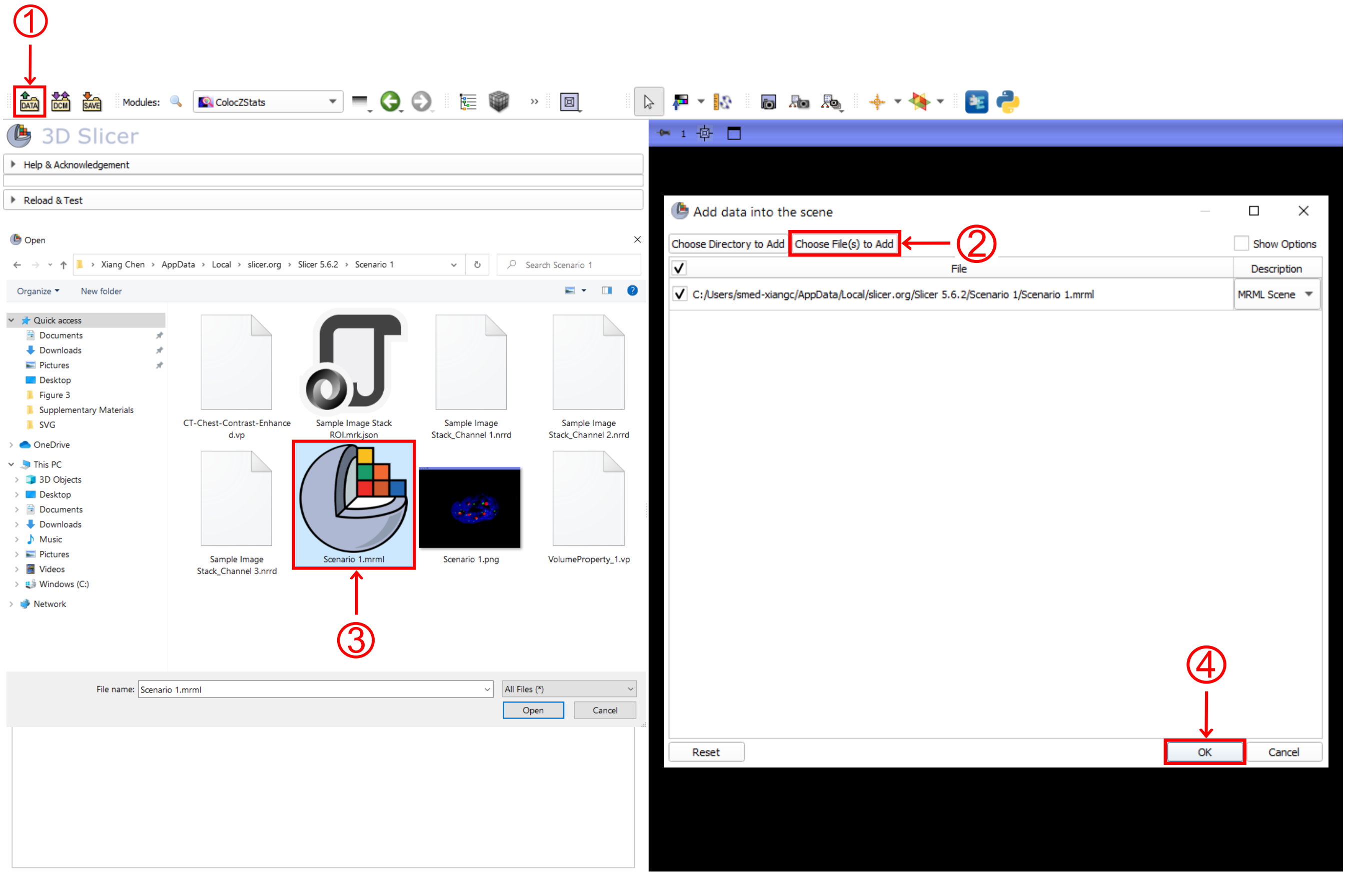

Figure S3. An illustration of loading 'Scenario 1.mrml' into ColocZStats. **(1)** Navigate to the ColocZStats extension and click on the 'DATA' button at the upper-left corner to open a pop-up window called 'Add data into the scene.' **(2)** Click the 'Choose File(s) to Add' button to open a file browser. **(3)** Navigate to the unzipped folder of 'Scenario 1.zip'and select the 'Scenario 1.mrml.' **(4)** Click the 'OK' button to load the MRML file into the scene.

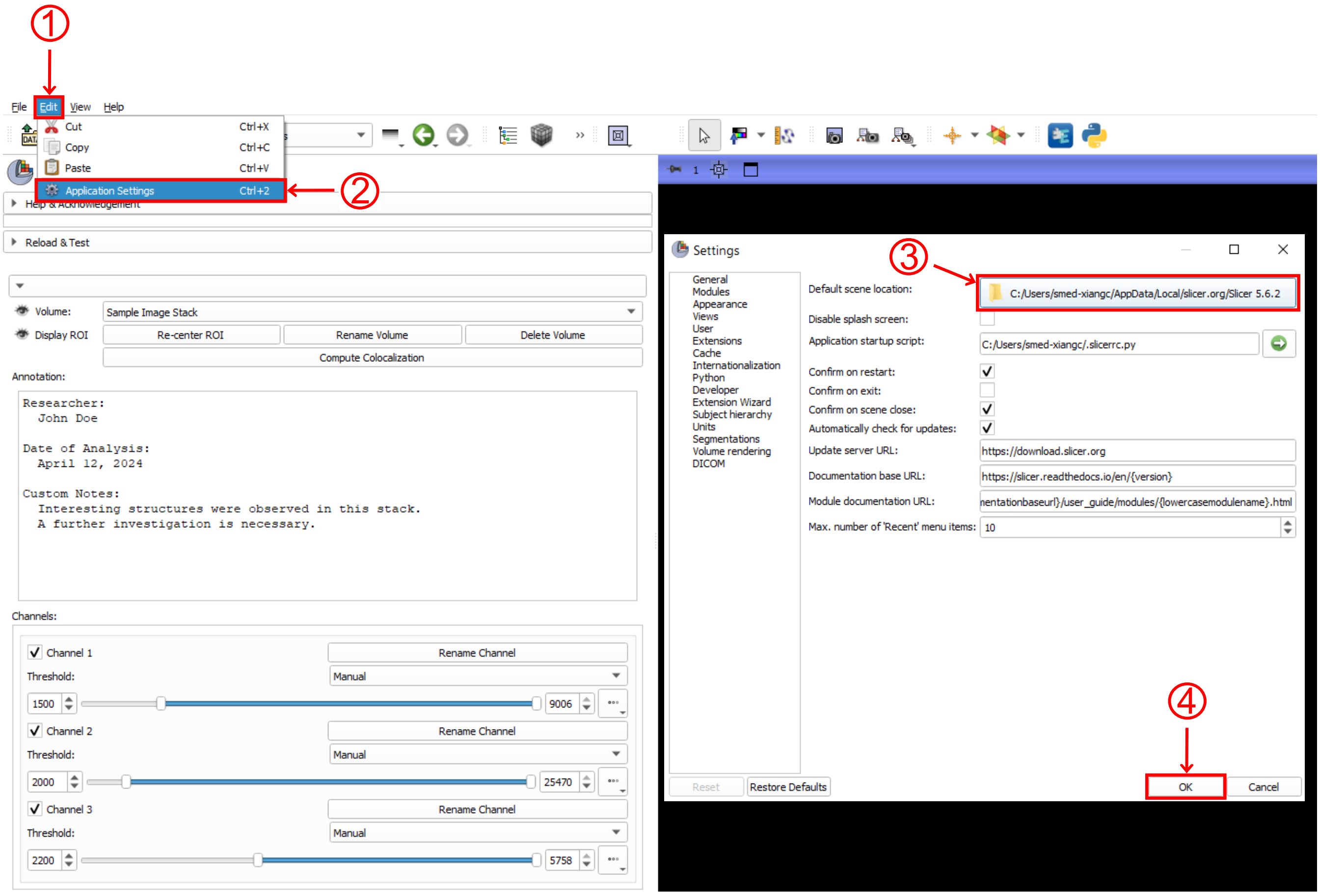

Figure S4. An illustration of specifying 'Default scene location' in 3D Slicer. **(1)** Click the 'Edit' button at the upper-left corner to open a drop-down list. **(2)** Click on the 'Application Settings' to open its pop-up window. **(3)** Click the button corresponding to the'Default scene location: ' to select a location with read and write permissions. **(4)** Click the 'OK' button to finish this setup.

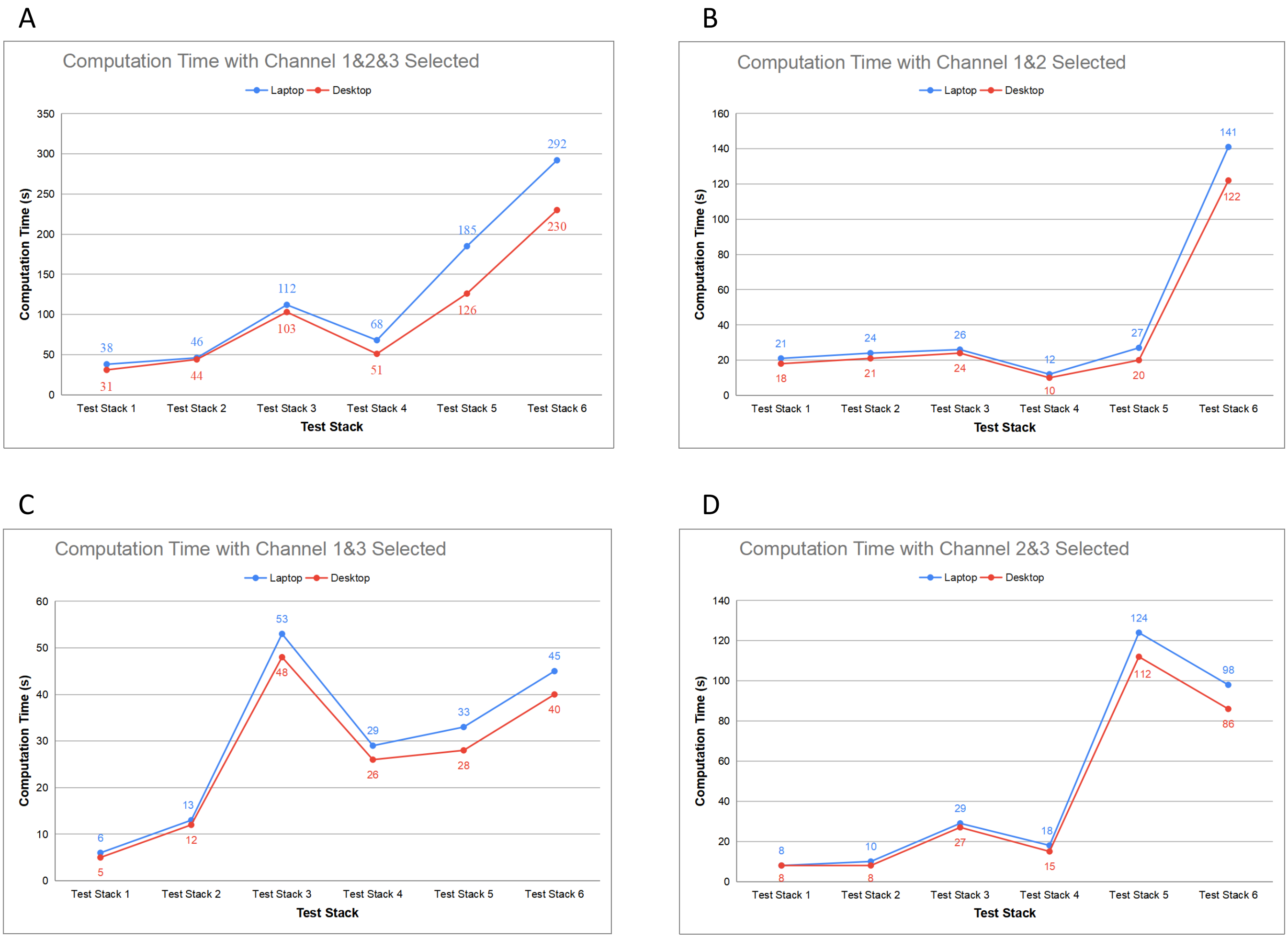

Figure S5. Computation Time Details. The sizes of the test stacks are as follows: Test Stack 1 (24.9MB), Test Stack 2 (60.0MB), Test Stack 3 (218.0MB), Test Stack 4 (355.0MB), Test Stack 5 (523.0MB), Test Stack 6 (679.0MB).
